# Emerging Epigenetic and Posttranslational Mechanisms Controlling Resistance to Glucocorticoids in Acute Lymphoblastic Leukemia

**DOI:** 10.1097/HS9.0000000000000916

**Published:** 2023-06-22

**Authors:** Cristina Borin, Tim Pieters, Valentina Serafin, Panagiotis Ntziachristos

**Affiliations:** 1 Department of Biomolecular Medicine, Ghent University, Belgium; 2 Center for Medical Genetics, Ghent University and University Hospital, Belgium; 3 Cancer Research Institute Ghent (CRIG), Belgium; 4 Department of Surgery Oncology and Gastroenterology, Oncology and Immunology Section, University of Padova, Italy

## Abstract

Glucocorticoids are extensively used for the treatment of acute lymphoblastic leukemia as they pressure cancer cells to undergo apoptosis. Nevertheless, glucocorticoid partners, modifications, and mechanisms of action are hitherto poorly characterized. This hampers our understanding of therapy resistance, frequently occurring in leukemia despite the current therapeutic combinations using glucocorticoids in acute lymphoblastic leukemia. In this review, we initially cover the traditional view of glucocorticoid resistance and ways of targeting this resistance. We discuss recent progress in our understanding of chromatin and posttranslational properties of the glucocorticoid receptor that might be proven beneficial in our efforts to understand and target therapy resistance. We discuss emerging roles of pathways and proteins such as the lymphocyte-specific kinase that antagonizes glucocorticoid receptor activation and nuclear translocation. In addition, we provide an overview of ongoing therapeutic approaches that sensitize cells to glucocorticoids including small molecule inhibitors and proteolysis-targeting chimeras.

## INTRODUCTION

### Glucocorticoids and their function in physiology

**Figure 1.**
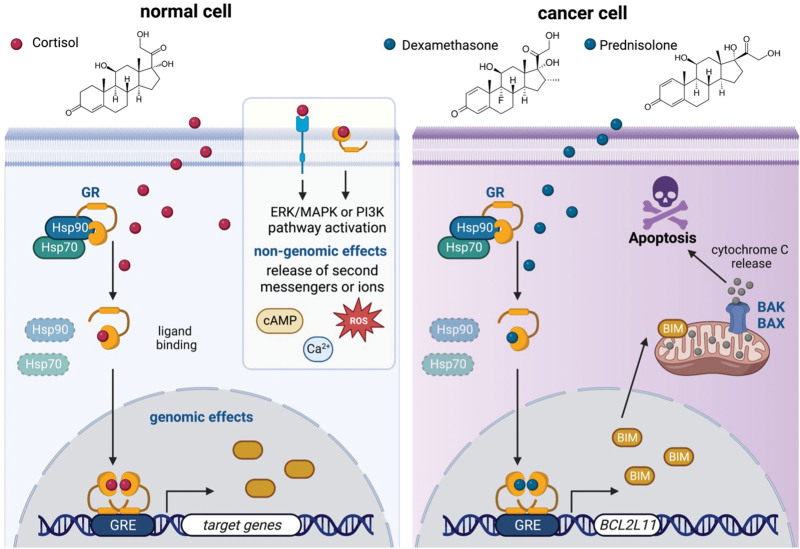
**GCs in normal physiology and anticancer therapy.** (Left) Humans endogenously produce GCs (eg, cortisol) in the cortex of the adrenal gland, and they can diffuse through the lipid bilayer and exert both genomic and nongenomic effects. The latter involves binding of membrane receptors (both steroid and nonsteroid), which induces rapid activation of kinases such as ERK, MAPK, and PI3K, as well as regulating the release of second messenger molecules such as cAMP, ROS, and intracellular calcium ions. The genomic effects of GCs require binding to the GR, which dissociates from cytosolic Hsp complexes, translocates to the nucleus, binds to GRE, dimerizes, and enables transcriptional activation or repression of GR target genes. (Right) Synthetic GCs, such as dexamethasone and prednisolone, are the cornerstone of lymphoid anticancer therapy and transcriptionally activate the *BCL2L11* gene, which encodes the proapoptotic BIM protein, which stimulates BAX and BAK oligomerization in the outer mitochondrial membrane, leading to cytochrome C release and caspase-dependent apoptosis of cancer cells. ERK = extracellular signal-regulated kinase; GC = glucocorticoids; GR = glucocorticoid receptor; GRE = glucocorticoid response elements; Hsp = heat-shock protein; PI3K = phosphatidylinositol 3-kinase; ROS = reactive oxygen species.

Glucocorticoids (GCs), such as cortisol, are steroid hormones that are endogenously produced in the adrenal cortex and that upon their release regulate multiple physiological processes, including fat metabolism, bone homeostasis, metabolism, and anti-inflammatory and immunosuppressive actions.^1,2^ GCs, through their lipophilicity, diffuse across the cell membrane to the cytosol, where they can exert both genomic and nongenomic effects (Figure 1, left). The nongenomic effects of GCs occur rapidly, in seconds or minutes, and influence the activation of kinases including mitogen-activated protein kinase (MAPK) and protein kinase C, and the release of second messengers, including cyclic adenosine monophosphate (cAMP), reactive oxygen species, diacylglycerol, or calcium ions.^3,4^ The genomic effects of GCs are mediated through binding to the glucocorticoid receptor (GR), belonging to the steroid family of nuclear hormone receptors, encoded by the *NR3C1* gene (nuclear receptor subfamily 3 group C member 1). The *NR3C1* gene resides on chromosome 5 (5q31) and comprises 9 exons, of which exons 2–9 encode the GR protein.

**Figure 2.**
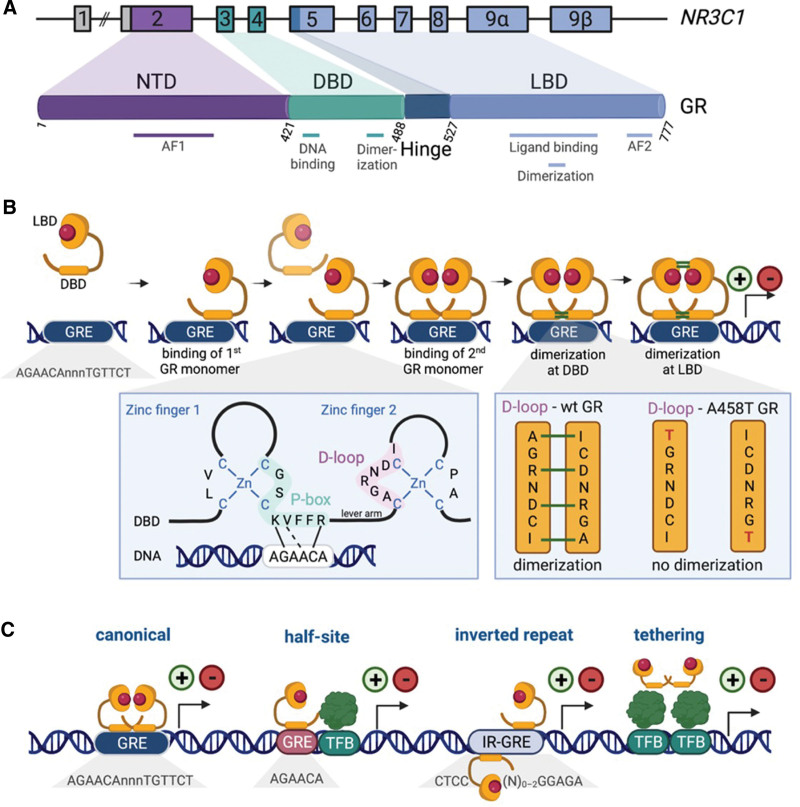
**Structure, dimerization, alternative modes of DNA-binding and transcriptional activity of GR.** (A) Structure of the human *NR3C1* gene and of the corresponding GR protein. Boxes denote exons, while cylinders represent protein domains. Exon 2 encodes the intrinsically disordered NTD, which harbors an AF1 domain. It is assumed that the NTD can adopt various conformations upon binding of different cofactors and, as such, may induce specific transcriptional responses. In addition, different translational start sites are present in the NTD and produce multiple NR3C1 isoforms. The DBD contains amino acid residues that are important for DNA binding and GR dimerization (see details in B). The LBD harbors amino acid residues that are involved in ligand binding, GR dimerization, and transcriptional activation (AF2). (B) Schematic representation of step-wise binding of GR monomers to a GRE, leading to sequential dimerization by interactions within the DBD and LBD. GR binding to canonical GREs occurs via 3 residues (Lys442, Val443, and Arg447) from the P-box (part of the first zinc finger of the DBD) that make base‐specific contacts within the major groove of the DNA, in the GRE area, by using either hydrogen bonds (black solid lines) or van der Waals interactions (black dashed line). A lever arm (residues 469–474) connects the P-box with the distal loop (D-loop) within the second zinc finger, and its conformation can be changed according to the type of DNA binding. Upon binding of the GR to an optimal canonical GRE, the lever arm will adopt a conformation that favors interaction between the D-loops of 2 GRE monomers, and will result in DBD dimerization. The D-loop interactions between opposing DBDs include a hydrogen-bond between A458 and I464, and salt bridges between R460 and D462. DBD dimerization triggers the dimerization of the LBDs of 2 GR molecules that are oriented in a head-to-head orientation. The LBD dimer interface is less conserved than the DBD dimer interface, and several alternate structures have been described. A458, R460, and D462 are crucial for proper dimerization, and mutants that affect them, such as A458T, severely impair GR dimerization between D-loops. (C) GR can interact as a dimer with the canonical GRE, but also as a monomer with a GRE half-site that typically contains a single-hexamer related to the GRE consensus sequence and may cooperate in conjunction with proximal non‐GR TFs (green). In addition, 2 GR monomers can bind in a head-to-tail fashion to IR-GREs present within the DNA. These monomers are bound to opposite sides of the DNA. Finally, the GR presents with indirect binding to genomic loci via protein-protein interactions to other transcription factors. All these transcriptional modes can activate (plus symbol) or repress (minus symbol) GR-responsive genes. AF1 = activator function; DBD = DNA-binding domain; GR = glucocorticoid receptor; GRE = glucocorticoid response elements; IR-GRE = inverted-repeat GREs; LBD = ligand-binding domain; NTD = N-terminal domain; P-box = proximal box; TFB = transcription factor binding site; TFs = transcription factors.

The GR is constitutively expressed and consists of an amino-terminal domain (NTD), a DNA-binding domain (DBD), a hinge region, followed by a ligand-binding domain (LBD) (Figure 2A). In its unbound state, the GR is localized in the cytoplasm, where it is sequestered by molecular chaperone complexes of heat-shock protein 90 (HSP90), HSP70, and other factors.^5^ Upon binding to endogenous GCs cortisol (humans) or corticosterone (rodents), GC-bound GR dimers translocate into the nucleus where they can bind to glucocorticoid response elements (GREs), activating or repressing genomic targets.^6,7^

### Dimerization and transcriptional modes of action of GR

The intracellular GR directs the transcriptional activity of multiple steroid-responsive genes, including its own locus *NR3C1*,^8^ via various modes, for example, by binding to DNA directly at canonical, half-site, or inverted-repeat GREs, or by indirectly binding to other DNA-bound transcription factors (TFs) (Figure 2B and 2C).

The best-characterized GR–DNA interaction is through the canonical GRE sequence AGAACAnnnTGTTCT, which is an imperfect palindrome comprising two 6-bp half-sites where GR binds as a monomer and then dimerizes (Figure 2B)^6,9^ This step-wise process starts when a GC-bound GR monomer enters the nucleus and binds to one of the half sites of a canonical GRE via its DBD. The DBD contains 2 zinc fingers that harbor a proximal box (P-box) and distal loop (D-loop), which are important for DNA binding and DBD dimerization, respectively. GR binding to DNA occurs via 3 P-box residues (K442, V443, and R447) that interact with nucleotides within the major groove of the canonical GRE DNA, using either hydrogen bonds or van der Waals interactions (Figure 2B). The 2 zinc fingers are connected by a lever arm (residues 469–474) that can alter its conformation depending on the type of DNA binding. Hence, the lever arm mediates high- or low-affinity binding to optimal and nonoptimal GREs, respectively, through allosteric modification. Upon binding of the GR to an optimal canonical GRE, the lever arm will adopt a conformation that favors interaction between the D-loops of 2 monomers and will result in DBD dimerization. The D-loop interactions between opposing DBDs include a hydrogen-bond between A458 and I464, and salt bridges between R460 and D462 (Figure 2B). D-loop residues A458, R460, and D462 are crucial for proper dimerization, as mutations that affect them, such as A458T, severely impair GR dimerization between D-loops.^10,11^

DBD dimerization triggers the dimerization of the LBDs of 2 GR molecules that are oriented head-to-head. The LBD dimer interface is less conserved than the DBD dimer interface, and several alternate structures have been described.^9^ Nevertheless, 2 important residues, namely P625 and I628, have been identified at the LBD dimer interface, and mutating either one of them to alanine results in a 10-fold decrease in dimerization affinity.^12^ Once fully dimerized, DNA-bound GR homodimers will exert their transcriptional activity by inducing both activation and repression of GC-dependent genes.

Besides binding of GR dimers to canonical GREs, alternative transcriptional modes that involve direct binding of GR to DNA exists, directly via half-sites or inverted-repeat GREs, or indirectly by binding to other DNA-bound TFs (Figure 2C).^6^ Like with canonical GREs, GR monomers can also induce selective binding to half-site DNA sequences (consensus AGAACA), close to other TF-bound sites to form a composite GR-TF complex. For a long time, it was believed that the undesirable side effects of GC therapy were induced by GR dimer-mediated transactivation, whereas its beneficial anti-inflammatory effects were mainly due to GR monomer-mediated gene transrepression.^13^ However, many reports using GR^dim/dim^ mice, which carry the A465T mutation (similar to A458T in humans), show that GR dimer-dependent transactivation is also essential for inducing the expression of anti-inflammatory genes.^9,14^ Recent ATACseq and ChIPseq data in GR-null cells that express wild-type or dimerization-defective (either A465T or A465T/I634A mutant) GR showed that only wild-type GR dimers can open closed nucleosomal sites and induce transcriptional activity at these sites.^15^

GR–DNA interaction can also happen through inverted-repeat GREs (Figure 2C), where GR molecules bind on opposite sides of DNA to canonical half-site DNA sequences (consensus AGAACA) or through a tethering mechanism that does not involve direct binding of GR to DNA, but where the DNA-binding domain makes protein-protein interactions with TFs such as activator protein 1 (AP-1) or nuclear factor-κB (NF-κB).^6^ According to the hit-and-run model, the loading of the GR in vitro happens transiently at the level of the promoter (the hit), and is then dynamically uncoupled from hormone response elements beyond 20 minutes (the run).^16^ Thus, in these binding modes, the receptor is not statically bound to the promoter but undergoes rapid exchange between chromatin and the nucleoplasmic compartment.^17^ Finally, recent evidence hints that the GR might also exist as a tetramer, or as a dimer of GR dimers.^18^

### GCs and their functions in the treatment of acute lymphoblastic leukemia

GCs are known to be effective in killing lymphoid cells. This was based on the observations that the size of the adrenal cortex, the source of GCs, is inversely correlated with the size of the thymus,^19^ and that administration of adrenocorticotropic hormone, which triggers cortisol release from the adrenal gland, reduced the volume of most lymphoid tissues.^20^ Due to their effectiveness against lymphoid malignancies, synthetic GCs, such as dexamethasone and prednisolone, have been deployed as the cornerstone of anticancer therapy for decades.^21^ These 2 synthetic analogs of cortisol differ in their molecular structure and in their pharmacokinetic profile^21^ (Figure 1, right). Dexamethasone has an extended plasma half-life and biological half-life, higher potential to penetrate into the central nervous system (CNS), and better control of CNS leukemia.^21^ Thus, the cytotoxic effect of dexamethasone is much greater than that of prednisolone in primary pediatric acute lymphoblastic leukemia (ALL) samples^22^ and in leukemia cells grown on bone marrow-derived stromal layers.^23^ The dexamethasone-GR complex is more stable than the prednisolone-GR complex,^24^ and dexamethasone treatment in ALL patients results in a better 6-year event-free survival rate and reduced bone marrow and CNS relapse compared with prednisolone.^25^ Comparison between dexamethasone versus prednisone in induction therapy in the context of Associazione Italiana di Ematologia e Oncologia Pediatrica and Berlin-Frankfurt-Munster pediatric ALL trial showed that there is significant relapse reduction and increased treatment-related mortality upon dexamethasone treatment.^26^ However, the benefit of dexamethasone was partially counterbalanced by a significantly higher induction-related death rate (2.5% [dexamethasone] versus 0.9% [prednisone]). This led to no overall survival change between dexamethasone and prednisone groups except in the subset of patients with T-cell ALL and good early treatment response.

The effectiveness of GCs is mainly due to GR-dependent transcriptional activation of proapoptotic genes, mainly the *BCL2L11* gene, which encodes the protein BIM (Bcl-2 interacting mediator of cell death). BIM counteracts the antiapoptotic activity of BCL2, BCL-XL, and MCL1 and induces BAK/BAX oligomerization and the formation of the mitochondrial pore, which in turn triggers mitochondrial cytochrome C release and the induction of caspase-dependent apoptosis in steroid-sensitive lymphoblasts (Figure 1, right).^15,16^

Despite the beneficial effects of GCs, they cause adverse effects as well, including infection, bone fracture, osteonecrosis, mood and behavior problems, and myopathy. For children over 10, and adults, dexamethasone is significantly more likely to cause avascular necrosis, psychiatric issues, muscle wasting, and mortality.^27^ Effects that arise years after the cessation of treatment are also a concern. The muscle wasting, osteoporosis, and metabolic effects of GCs can persist after treatment ends, and the eventual neuropsychiatric effects are an emerging symptom to consider. More recently, it has been shown that pulsing dexamethasone during maintenance can produce the same outcomes with more acceptable side effects. Nonetheless, physicians are currently challenged with the choice of which GC to use, at what dose, and for how long. A more complete understanding of GC biology and function in leukemic blasts and other tissues will help inform these choices.

In this review, we discuss the established and emerging mechanisms of action of GCs in ALL, as well as associated mechanisms of therapy resistance (see Table 1). ALL is a blood cancer characterized by the rapid proliferation of abnormal lymphocytes that can arise from B (B-ALL) or T (T-ALL) cell precursors. Despite the effectiveness of GCs in reducing lymphoid disease, as single agents they did not produce durable remission, much less a cure. The current standard of care for acute leukemia entails intensive induction chemotherapy, consisting of a GC (prednisone or dexamethasone), vincristine, an asparaginase preparation, optional use of an anthracycline, and intrathecal chemotherapy. Pediatric T-ALL cases are typically more aggressive than B-ALL cases showing resistance to the first 7 days of GC treatment. These patients with poor response after 1 week of GC treatments (>1000 blasts/mm^3^ prednisone-poor responders [PPR]) are classified as high-risk patients. PPR T-ALL that show ex vivo resistance to GCs have a significantly worse treatment outcome (disease-free survival) than patients whose cells are sensitive to GCs^22,47^ and receive a high-risk adapted therapy. Even in this case, they respond poorly compared with other high-risk patients.^48,49^ This underlines the unmet clinical need for the treatment of PPR T-ALL cases.

**Table 1:** Mechanisms of Resistance to GCs and Associated Therapeutic Interventions

Resistance Mechanism	ALL Type	Incidence	Potential/Suggested Intervention	References
Genetic				
NR3C1 mutations	B-ALL, T-ALL	10%–12% of ALL cases, enriched in relapsed cased and ETV6/RUNX1 leukemia cases		28–30
IL7 mutations or otherwise hyperactivation (via NOTCH1)	T-ALL	32% of T-ALL cases	IL-7 antibodies, MEK/ERK inhibitors (eg, selumetinib), JAK inhibitor (ruxolitinib), PIM1 inhibitors	31–34
NSD2 mutations	B-ALL	14% of B-ALL cases (E1099K), enriched ETV6/RUNX1 and at relapse	PRC2 inhibitors	35,36
CBP mutations	B-ALL	18.3% of relapsed B-ALL cases (might be present at diagnosis too)	HDAC inhibitors	37
Splicing				
NR3C1 splicing (altered isoform balance)	B-ALL, T-ALL		Antisense oligos blocking NR3C1 splicing (?)	38
** **Signaling and posttranslational regulation				
TCR and LCK activity	T-ALL		LCK inhibitors (dasatinib) and USP inhibitors	39
PI3K	B-ALL, T-ALL		PI3K inhibitor (LY294002)	31,32,40–42
mTOR activity	T-ALL		mTOR inhibitor (temsirolimus) and dasatinib	43
SGK1 activity	T-ALL		SGK1 inhibitors (eg, GSK650394)	44
AURKB activity	B-ALL		AURKB inhibitors (eg, ZM447439)	45
USP7/USP11/LCK activity	T-ALL		USP7 inhibitors (eg, P5091)	46

ALL = acute lymphoblastic leukemia; AURKB = aurora kinase B; CBP = cAMP response element-binding protein; ERK = extracellular signal-related kinase; GC = glucocorticoid; LCK = lymphocyte-specific protein tyrosine kinase; MEK = MAPK kinase; mTOR = mammalian target of rapamycin; NOTCH1 = Neurogenic locus notch homolog protein 1; SGK1 = serum and GC-inducible kinase-1; TCR = T-cell receptor.

### Established mechanisms of resistance to GCs

#### Genetic and nongenetic alterations of the NR3C1 locus affecting response to GCs

GC response is variable among T-ALL patients, and steroid resistance has been observed in a significant fraction of patients during induction therapy. Secondary resistance to GCs might lead to therapy failure.^50^ In this section, we will describe genetic alterations at diagnosis and relapse in the *NR3C1* locus that can lead to GC resistance.

**Figure 3.**
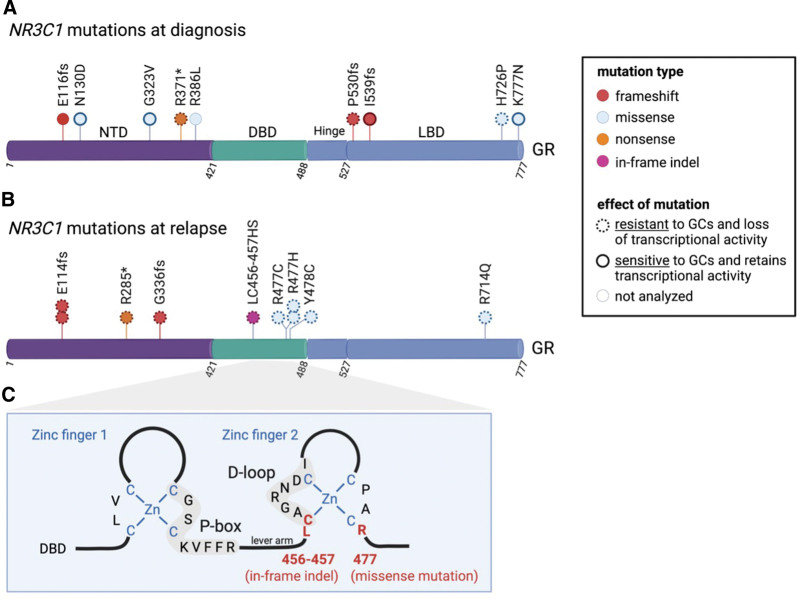
**NR3C1 genetic alterations in ALL patients at diagnosis and relapse.** (A) and (B) Structure of the human GR protein, consisting of a NTD, a DBD, and a LBD. Alterations in the GR protein due to frameshift (red), missense (blue), nonsense (orange), or in-frame indels (pink) mutations in the *NR3C1* gene are indicated. Dashed circles denote that this mutation causes resistance to GCs and loss of transcriptional activity, while full thick lines denote that this mutation does not induce resistance to GCs and retains normal transcriptional activity. (C) Blow-up from the DBD of the GR, showing 2 zinc fingers, the P-box and D-loop. Residues in the second zinc finger that are affected by an in-frame indel or by a recurrent missense mutation are shown in red. ALL = acute lymphoblastic leukemia; DBD = DNA-binding domain; D-loop = distal loop; GR = glucocorticoid receptor; LBD = ligand-binding domain; NTD = N-terminal domain; P-box = proximal box.

At diagnosis, *NR3C1* alterations are rare in ALL. Three studies report *NR3C1* mutations in 0.9% (3/333),^51^ 1.9% (2/103),^52^ and 3% (5/146; juvenile T-ALL cohort)^53^ of patients with newly diagnosed ALL. These include heterozygous missense, frameshift, and nonsense mutations (Figure 3A). These mutations are dispersed over the entire GR protein, but their impact on transcriptional activity and therapeutic outcome is variable.^51,53^ In addition to point mutations, also deletions of the long arm of chromosome 5, which encompass the *NR3C1* gene, were observed in 4% (6/146)^53^ and 8% (17/200)^54^ of T-ALL patients at diagnosis. Of note, 5q deletions were predominantly found in early T-cell precursor (ETP)-ALL and HOXA + T-ALLs.^53,54^ At diagnosis, *NR3C1* deletions were also observed in 13% (6/47)^28^ of childhood *ETV6/RUNX1* ALL. In conclusion, *NR3C1* aberrations are relatively rare in newly diagnosed ALL, and although patients with either *NR3C1* mutations or deletions have a significantly inferior response to GCs in vitro compared with *NR3C1* wild-type patients,^53^ the response to GCs and the transcriptional activity in ALL patients with *NR3C1* alterations is variable.

In a second phase, at relapse, ALL patients frequently present acquired therapy-induced *NR3C1* alterations.^29,30,37,51,52,55–57^ Initially, a L793F mutation was found in a GC-resistant CEM cell line and this mutation could be traced back to the original lymph node biopsy from which the CEM cell line was derived.^55^ Moreover, *NR3C1* mutations or deletions were recurrently observed in 2% (1/50; Δ702),^56^ 9% (2/23; 5q deletions),^37^ 11% (2/18; R477H and Y478C),^51^ 12% (12/103; missense and frameshift mutations),^52^ or 9% (18/238; deletions)^57^ of relapsed ALL patients that were treated with GCs (Figure 3B). Strikingly, all the *NR3C1* mutants that were functionally analyzed showed a reduced transcriptional activity and were resistant to GC treatment.^51,52^ Several *NR3C1* mutations, including a recurrent mutation that affects R477, cluster around zinc finger 2 of the DBD of GR (Figure 3C), and probably interfere with GR-driven transcription by preventing proper GR dimerization. Mutations that affect GR residue R477 were also described in patients with primary cortisol resistance^58^ and GC-resistant ALL cell lines.^59^ Of note, depending on the type of amino acid substitution, the R477 mutant can either act as dominant negative or not.^51^ Also, de novo *NR3C1* deletions were identified in 28% (4/14),^28^ 10% (5/51),^29^ or 16% (5/31)^30^ of relapsed childhood *ETV6/RUNX1* ALL, which were associated with poor response to GC treatment. This implies that ALL patients, who are treated with a GC-containing chemotherapy regimen, are actively selecting for *NR3C1* alterations that confer resistance to GC treatment. This acquired resistance to GC treatment was further confirmed in an experimental model for therapy-induced relapse in T-ALL patient-derived xenografts (PDXs).^60^ To do so, primary T-ALL bone marrow samples were transplanted in immunocompromised mice and were treated with repeated blocks of a 4-drug chemotherapy combination (vincristine, dexamethasone, L-asparaginase, and daunorubicin), which closely resembles the current ALL induction-therapy protocols. In this model, Yadav et al^60^ found that T-ALL cells acquired resistance to dexamethasone, while they remained sensitive to the other 3 chemotherapeutic agents. Moreover, no common gene expression changes were observed among different dexamethasone-resistant relapsed PDX models, suggesting that mechanisms of resistance might differ among individuals.^60^ In conclusion, GC treatment induces selection toward loss-of-function *NR3C1* mutations in ALL. However, genetic inactivation cannot explain all cases of steroid resistance, indicating that there are other nongenetic mechanisms that confer steroid resistance, in line with an early study using the GC-resistant T-ALL cell line CEM and a GC-sensitive clone identified no mutations in the N3RC1 locus.^61^

The role of the actual GR levels in GC response has been investigated; negative regulation of NR3C1 levels in response to high GC concentration has been described early on.^62^ *NR3C1* is known to be positively regulated at the transcriptional level by GR itself, and it is also negatively regulated by the Neurogenic locus notch homolog protein 1 (NOTCH1) target hairy and enhancer of split-1 (HES1) that antagonizes GR binding to NR3C1 control elements; HES1 binds and suppresses the *NR3C1* promoter.^63^ GC resistance in ALL may also be caused by an increase in the expression of GR variants resulting from alternative splicing or translational initiation.^64–66^ The 2 main isoforms, namely GRα and GRβ, are generated via alternative usage of exon 9α and exon 9β, respectively (Figure 2A). GRα is considered the prototypic and most common GR isoform, while GRβ has a shorter ligand-binding domain and is unable to bind GCs, but it might contribute to GC resistance by competing with GRα for DNA binding.^38^ Other studies, however, showed that in vitro sensitivity to GCs does not depend on the proportion of GRα-to-GRβ levels.^67^ Additionally, GRα mRNA levels per se have not been associated with resistance to GCs, although resistant cases might present with poor transcript translation.^67^ It was demonstrated that NF-κB, an oncogenic TF in T-ALL, binds DNA to the 5′ side of the GR promoter, causing a disproportionate increase in the GRβ protein isoform over GRα. In the same way, IL-1β, a proinflammatory cytokine that activates NF-κB, induces an increase in GRβ levels.^68^ Studies on other variants such as GRγ and GR-P showed that their expression can have a role in changing GC sensitivity in hematological malignancies. GRγ acts as a TF, with 50% of the activity of GRα, and its expression has been linked to resistance to dexamethasone treatment in ALL,^69,70^ as well as resistance in patients with small-cell lung carcinoma and corticotrope adenomas.^71^ Cells from patients with a good response to prednisone display higher levels of apoptosis and lower GRγ expression compared with cells from patients with a PPR.^70^ In addition, multiple GR isoforms can be produced by using different translational start sites that are present in the NTD and these isoforms might affect their transcriptional activity and response to GCs.^66^

#### GR is antagonized by several oncogenic pathways that lead to therapy resistance

Recently, it became clear that resistance to steroids is mediated through altered signaling pathways rather than isolated mutations (see Table 1). For example, some studies have suggested that activating mutations in the Janus kinase (JAK)-signal transducer and activator of transcription (STAT) pathway, downstream of GCs can lead to resistance to GC therapy in ALL.

NOTCH signaling is dysregulated in about 60% of T-ALL patients.^72–74^ T-ALL patients carrying NOTCH mutations represent a subset of NOTCH-addicted T-ALL who will be treated with specific drugs targeting the NOTCH signaling pathway. Interleukin 7 (IL-7) cooperates with NOTCH to promote GC resistance. Physiological activation of the pathway is induced when IL-7 binds to its cognate receptor, which results in the activation of downstream JAK/STAT and phosphatidylinositol 3-kinase (PI3K)/AKT pathways.^40^ In T-ALL, IL-7 signaling can be activated both due to IL-7 receptor mutations or after IL-7 stimulation that induces GC resistance through the upregulation of MAPK kinase (MEK)/extracellular signal-related kinase (ERK) and PI3K/AKT/mammalian target of rapamycin (mTOR) pathways, which can in turn be reversed by IL-7 and/or MEK/ERK inhibitor treatment.^31,32,41^

A study using 146 pediatric T-ALL patients discovered the presence of mutations of IL-7 pathway genes in 47 (32%) samples associated with resistance to GC treatment and poor outcome.^32^ Those mutations alone, or when stimulated with IL-7, caused activation of the PI3K-AKT pathway, resulting in an increase of antiapoptotic proteins, such as BCL2, MCL-1, and BCL-XL levels, and a decrease in the GC-induced proapoptotic BIM protein, which all contribute to steroid resistance.^31,32^ Moreover, the MEK inhibitor selumetinib synergizes with steroids in both IL-7-dependent and IL-7-independent steroid resistant pediatric T-ALL PDX samples.^32^ Upregulation of BCL2 and BCL-XL in STAT5-activated T-ALL cells require steroid-induced activation of *NR3C1* coupled to GR binding to BCL2 and BCL-XL control elements.^38^ An *shRNA* screen in B-ALL identified PI3K delta (PI3Kδ) as a critical factor in GC-response and resistance to therapy. Inhibition of PI3Kδ, a component of the pre-B-cell receptor and IL-7 receptor signaling pathways critical to B-cell development using idelalisib, enhances the effect of GC and GR activation on transcription to push GC-resistant B-ALL to apoptosis.^42^

The T-cell receptor (TCR) and its downstream Ca^2+^ signaling cascade are critical in the activation and survival of normal T cells.^75^ T-ALL cell proliferation relies on this altered Ca^2+^ signaling cascade, independently of TCR antigen binding. TCR signaling involves the downstream phosphorylation of the lymphocyte-specific protein tyrosine kinase (LCK) that induces a sustained Ca^2+^ influx via the plasma membrane Ca^2+^ channel (CRAC) and the activation of calmodulin by Ca^2+^ binding, which in turn activates the calcineurin phosphatase that dephosphorylates the nuclear factor of activated T-cells (NFAT) TF family, allowing their import into the nucleus and a consequent initiation of transcriptional programs.^76,77^ LCK hyperactivation at diagnosis has been associated with T-ALL PPR status in ETP-ALL patients^39,78^ as well as with PAX5 translocation-related poor prognosis in B cell precursor (BCP)-ALL subgroups.^79^ NFAT family members have been widely described as crucial for many aspects of the immune response and for the maintenance of the T-ALL phenotype.^80,81^ Physiologically, GCs can modulate intracellular Ca^2+^ levels by inducing the expression of serum and GC-inducible kinase-1 (SGK1), which is involved in tumor growth and resistance to GC chemotherapy. Nedd 4-2/14-3-3 phosphorylates SGK1, leading to inhibition of Orai-1 ubiquitination, the main channel-forming subunit of CRAC,^44^ that might result in enhanced CRAC activity and T-ALL survival.

Hedgehog pathway mutations and aberrant signaling in T-ALL are observed in ~20% of T-ALL cases.^82^ Most of the approved drugs work on the smoothened receptor, but recent studies have also identified the GLI1 TF as an important target of choice in T-ALL.^82^ Bongiovanni et al^83^ proposed targeting the crosstalk between Hedgehog signaling and the GR pathway as an effective therapeutic strategy in T-ALL. This derives from a synergistic antileukemic effect observed in T-ALL cell lines and in PDX models upon combinatorial treatment with the GLI inhibitor GANT61 and dexamethasone. In the proposed mechanism, dexamethasone-induced *NR3C1* expression can favor the recruitment of PCAF acetyltransferase and the dissociation of HDAC1 deacetylase, leading to GLI1 acetylation and impairment of GLI1 transcriptional activity and protein stability.^83^

Inactivating mutations or deletions in the negative regulator phosphatase and tensin homolog (PTEN), PI3K subunits, or AKT1 have been extensively described in pediatric T-ALL patients.^84^ Specifically, the role of PTEN in GC resistance is still debated in the scientific community. Indeed, in the ALL IC-BFM study protocol,^85^ genomic alterations in PTEN have been associated with poor response to corticosteroid therapy, unfavorable clinical outcomes, and an augmented risk for relapse. However, this association between PTEN alterations and poor clinical outcome has not been further confirmed in the UKALL2003 trial.^86^ Conversely, the direct phosphorylation of the GR by AKT, which blocks its translocation into the nucleus, has been deeply investigated by Piovan and colleagues.^87^

The Rat sarcoma virus (RAS)/MEK/ERK signaling is mutated in many cancers, including hematological malignancies, although the exact association between RAS/MEK/ERK signaling and therapy resistance remains to be clarified. Physiologically, H-RAS, N-RAS, and K-RAS are small GTPases that are active when bound to GTP and inactive when bound to GDP. Active RAS signals through Raf kinases, which trigger MAPK activation, culminating in the activation of ERK1/2. Specifically, in T-ALL patients, there are activating mutations in *N-RAS*, *K-RAS*, or *BRAF*. These kinases subsequently phosphorylate TFs such as Fos and Jun, which dimerize to form the AP-1 TF.^88^ In addition, loss-of-function alterations targeting *NF1* or *PTPN11* or activating mutations in FLT3, EGFR, or the IL-7 receptor have also been identified as alternative mechanisms for downstream MEK/ERK pathway activation.^32,84,89^

The *BTG1* gene was shown to be recurrently deleted in pre-B-ALL, and this renders pre-B-ALL cells refractory to the apoptosis-inducing effects of GCs. *BTG1* silencing in RS:11 induced a 10-fold reduction of GR expression, and this seems to be in part due to defective GR autoinduction. However, increasing GR or BTG1 expression did not fully restore therapy response.^90^ Other mutations have been found to increase GC resistance in B-ALL. For instance, loss-of-function mutations of the IKAROS family zinc finger 1 partially overlap with the GR-binding sites, affecting GR-dependent transcription and GC-mediated cell death.^91^ Also, TBL1X receptor 1 silencing results in attenuated GC signaling because of reduced GR recruitment to GC-responsive genes death.^92^

Molecular and functional analysis in B-ALL identified the aurora kinase B (AURKB) as a critical player controlling resistance to GCs. AURKB is highly expressed in relapsed disease cases and is a negative regulator of the GR coactivator complex comprising EHMT1 (also known as GLP), EHMT2 (also known as G9a), and CBX3.^45^ This kinase phosphorylates EHMT1-2 blocking GC-mediated gene activation, independently of its role in cell cycle regulation. Inhibition of AURKB enhanced GC-mediated apoptosis in relapsed B-ALL samples.

GC resistance has been associated with upregulation of the genes involved in glucose metabolism and increased glucose uptake into cells.^93–95^ One of the best-known mechanisms is the overexpression of the P-glycoprotein (P-gp), a member of the ATP-binding cassette transporter family. P-gp is expressed on the cell surface and is responsible for actively pumping a wide range of drugs, including GCs, out of the cell. Overexpression of P-gp can, therefore, reduce the intracellular concentration of GCs, making the cells less sensitive to their effects.

#### Current combinatorial therapeutic efforts including GCs in ALL

These discoveries have led to combination schemes using GCs and other compounds that present significant therapeutic benefits in certain cases, as it is outlined below.

##### NOTCH1 inhibitors

Gamma secretase inhibitors (GSIs) against NOTCH1 and GCs benefit each other. The use of GSIs leads to a reduction of the levels of NR3C1 transcriptional repressor and restored GC receptor self-activation, leading to reversal of resistance to GCs.^96^ Nevertheless, various GSIs evaluated in clinical trials have had on-target toxicities due to pan-NOTCH signaling inhibition, hampering the clinical applications of those compounds against relapsing disease cases.^97^ GCs-induced cyclin D2 (Ccnd2) expression prevents goblet cell metaplasia stimulated by inhibition of NOTCH signaling upon GSI treatment, suggesting that combination of GCs with GSI might have therapeutic benefits. Efforts have focused on antibodies targeting NOTCH1 (OMP52M51)^98^ or against its ligands^99–101^ as well as a pan-NOTCH inhibitor, CB-103,^102^ which blocks the NOTCH transcriptional complex and presents with a more tumor-specific effect. Another approach exploited to inhibit NOTCH1 activity is inhibition of the sarco/endoplasmic reticulum calcium ATPase using apsigargin.^103,104^ Potential combinations of those new anti-NOTCH therapies with GCs might benefit GC-resistant T-ALL patients. NCT02518113 evaluated the combination of NOTCH1 inhibitor Crenigacestat, in association with dexamethasone in adult patients with relapsed/refractory (R/R) T-ALL/T-cell lymphoblastic lymphoma (LBL), unfortunately demonstrated limited clinical activity at the recommended dose.^105^

##### IL-7 receptor pathway inhibitors

More recently, it has also become clear that noncell autonomous mechanisms could render leukemic T cells insensitive to GC therapy.^31,106^ The IL-7 receptor pathway represents a potential therapeutic target that has been widely shown to be involved in dexamethasone sensitization upon its inhibition. In patients with IL-7-dependent T-ALL, the combination of dexamethasone with the FDA-approved specific JAK1/2 inhibitor ruxolitinib has been proposed to reverse GC resistance, because it alters the balance between proapoptotic and antiapoptotic factors.^31^ Alternatively, IL-7 receptor blockade with a specific monoclonal antibody^107^ or the use of KZR-445, which inhibits IL-7 receptor translocation into the secretory pathway, attenuates the dexamethasone‐induced increase of cell-surface IL‐7 receptor and helps overcome IL‐7‐induced dexamethasone resistance.^33^ The IL-7/STAT5 pathway correlates with PIM1 activation in both T-ALL and T-cell leukemia and lymphoma, and PIM1 mediates cellular activities through the phosphorylation of several substrates, including MYC.^34^ Novel PIM inhibitors have been proposed in combination with GCs for the treatment of these diseases.^108^ In 2023, the NCT05745714 trial is open for adolescents and young adults (AYA) with R/R ALL and LBL, evaluating the safety and efficacy of ruxolitinib and venetoclax in combination with dexamethasone together with cyclophosphamide and cytarabine in children and AYA with R/R ALL/LBL whose tumor present with alterations in the IL7R/JAK-STAT pathway.

##### RAS/MEK/ERK pathway inhibitors

Although it is still not clear what the exact association is between activated RAS/MEK/ERK signaling and poor clinical outcome or GC resistance in T-ALL, there has been a substantial effort toward targeting this pathway. Indeed, the synergistic effect of selumetinib, a MEK1/2 inhibitor, with GCs has recently been described by Matheson and colleagues,^109^ who showed that this drug has excellent activity in those leukemias with RAS pathway mutations. Additionally, the results of the phase 2 clinical trial in which the novel combination of selumetinib and dexamethasone was tested in both adult and pediatric R/R ALL patients revealed that this drug combination may serve as a bridging treatment option for patients awaiting CAR T-cell therapy and a treatment option for relapses once CAR-T cell treatment has failed.^110^

##### AKT/mTOR pathway inhibitors

T-ALL patients carry inactivating mutations or deletions of the negative regulator PTEN, PI3K subunits, or AKT1 that lead to aberrant activation of PI3K signaling.^84^ Specifically, Gu and colleagues^111^ clearly demonstrated the capability of rapamycin, an mTOR inhibitor, to synergize with dexamethasone in T-ALL cells from pediatric patients. Similarly, Batista and colleagues^43^ observed that the mTOR signaling was highly activated in all T-ALL patients and further increased in vitro by stimulation with IL-7 normally produced by the bone marrow. Thus, they further demonstrated that rapamycin synergizes with dexamethasone treatment and that the addition of a PI3K inhibitor (LY294002) markedly potentiated the antileukemia effects of dexamethasone in pediatric T-ALL. Analysis of a wide array of primary human T-ALLs and PDX human:mouse models suggests that the combination of temsirolimus and dasatinib treatment will be efficacious for a large fraction of human T-ALLs.^112^ In light of this data, several phase 1 and 2 clinical trials in T-ALL have been started to test mTOR inhibitors in R/R T and B-ALL patients in combination with corticosteroids and other drugs used during the chemotherapy regimen (NCT01403415, NCT03740334, NCT01614197, NCT01184885). However, these compounds have not passed the clinical trial phase yet.

### Emerging players in GC biology

#### The role of GR protein folding, homeostasis, and posttranslational regulation in GC response and resistance

**Figure 4.**
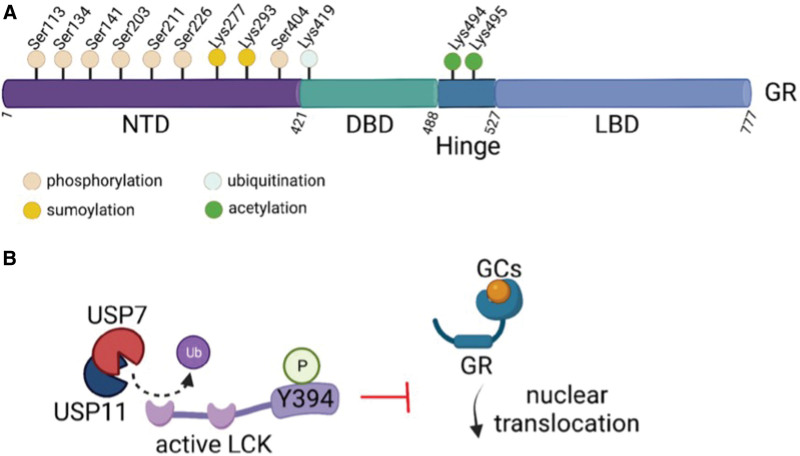
**Posttranslational modifications of the GR and LCK.** (A) Main sites of phosphorylation, ubiquitination, sumoylation, and acetylation on the GR. (B) Deubiquitination of LCK by the USP7/11 complex maintains LCK in its activated (Y394-phosphorylated) form that inhibits nuclear translocation of GR upon GC binding. GR = glucocorticoid receptor; LCK = lymphocyte-specific protein tyrosine kinase.

Recent research developments have demonstrated that there is extensive posttranslational regulation of the GR that might affect interaction, localization, and activity of GR and warrants further investigation (Figure 4). GR protein is regulated at the posttranslational level via ubiquitination, phosphorylation, and acetylation that control the interactome, nuclear translocation, and genomic binding of GR.^113–115^

##### Ubiquitination

Ubiquitin molecules covalently attach to Lys residues of proteins as single units or multimeric chains to affect protein localization, interactions, and stability via proteasomal degradation. Cells treated with proteasome inhibitors showed enhanced GR regulatory activity^113^ and increased in vivo DNA occupancy time, indicating that mechanisms such as ubiquitination might be involved in regulating GR stability. Upon disruption of the proteasomal activity, fluorescence recovery after photobleaching studies revealed a slow recovery of GFP-GR fusion protein at the mouse mammary tumor virus (MMTV) promoter, suggesting that proteasomal degradation is at least one of the means controlling GR levels.^116^ Chaperones and proteasomes are specifically recruited to the MMTV site and are required for dynamic GR interaction at the level of the promoter.^116^ GR ubiquitination at Lys419 was shown to stimulate GR nuclear export and subsequent degradation.^117,118^

Malyukova et al^119^ showed that loss of function of the E3 ligase FBXW7 may contribute to the malignant phenotype in T-ALL, but it subsequently works as a sensitizer to GC treatment. FBXW7 functions as a tumor suppressor by targeting oncoproteins for proteasomal degradation^120^ and its inactivation has a critical role in the development of T- ALL. On the contrary, the presence of *FBXW7* mutations in T-ALL identifies a subgroup of patients that exhibit a good response to GC chemotherapy and a more favorable outcome.^121,122^ This is explained by the fact that FBXW7 interacts with GRα through a conserved glycogen synthase kinase 3 (GSK3) β-phosphorylated degron, mediating GRα ubiquitination and proteasomal degradation. Inactivation of FBXW7 in leukemic cells enhances GRα stability and activity, promoting the transcriptional activity of GR-responsive target genes, including proapoptotic ones. This enhances GC sensitivity, demonstrating that inactivation of FBXW7 leads to elevated sensitivity to the cytostatic effects of GCs in T-ALL.^121,122^

##### Sumoylation

GR is tagged by the ubiquitin-like protein SUMO in 3 positions: Lys277, Lys293 (NTD), and Lys703 (LBD). These modifications affect GR transcriptional activity. Sumoylation has most commonly been linked to transcriptional repression.^123,124^ GR sumoylation mutants result in enhanced DNA recruitment for the transcriptional control of cell cycle and survival.^125^ Lys293 sumoylation might promote the assembly of repressive complexes, such as the silencing mediator of retinoic and thyroid (SMRT) receptors and nuclear receptor corepressor-containing ones.^126^ GR sumoylation might also promote GR binding to weaker associated sites and so loss of this sumoylation site results in diminished GR-regulated repression, via the absence of recruitment of repressive complexes.^126^

##### Phosphorylation

In addition to ubiquitination and sumoylation, GR cytoplasmic localization seems to be sensitive to phosphorylation, with 7 highly conserved amino acids on the NTD of the GR being targets for phosphorylation: Ser113, Ser134, Ser141, Ser203, Ser211, Ser226, and Ser404.^127^ Phosphorylation can be critical for protein localization and interactions, and these events lead to an increase in GR half-life, whereas phospho-mutant versions of the protein are prone to degradation.^128,129^ GR phosphorylation is the footprint of several oncogenic kinase pathways, including cyclin-dependent kinases, MAPKs, JUN N-terminal kinases (JNKs), and GSK-3.^130^ Phosphorylation of Ser203, Ser211, and Ser226 might affect cofactor interactions or enhance recruitment of the GR to genomic targets.^131–133^ There is crosstalk between those sites, as mutation of Ser203 prevents phosphorylation at Ser226.^134,135^ Ser203 is phosphorylated upon binding of GC, leading to GR nuclear translocation, whereas Ser226 phosphorylation, induced by JNK pathway activity, leads to nuclear export of GR, antagonizing GR transcriptional activities.^135,136^ Loss of Ser404 phosphorylation changes the conformation of GR, alters cofactor recruitment and transcriptional responses, and promotes GC-induced apoptosis.

Piovan et al^87^ discovered that AKT1 interacts with and directly phosphorylates GR at Ser134 to block GC-induced GR translocation to the nucleus, driving GC resistance in T-ALL. In this study, it was also shown that loss of PTEN and consequent AKT1 activation can block GC-induced apoptosis and induce resistance to GC therapy. In addition, pharmacologic inhibition of AKT using MK2206 was able to restore GR translocation to the nucleus, thus increasing response to GC therapy.^87^ AKT1 cooperates with 14-3-3 phospho-serine/threonine-binding proteins to affect GR transcriptional activity. Initial GR phosphorylation at Ser134 and subsequent association of 14-3-3 leads to tethering of GR in the cytoplasm, whereas the second phosphorylation event leads to enhanced GRα transcriptional activity directly in the nucleus. In this last event, AKT1 and 14-3-3 are attracted in a dexamethasone-dependent way to GREs of the MMTV promoter, followed by AKT1-mediated phosphorylation of p300 at Ser1834 (stimulating its histone acetyltransferase activity) and the tail of histone H3 at Ser10, which provide a binding site for 14-3-3. The outcome is an increase in the acetylation of the histone tails and a stimulation of the transcriptional activity of downstream targets.^137^

The information presented above suggests that further studies are warranted to understand GR posttranslational regulation and crosstalk between the different modifications.

##### Chaperones and other factors

In the absence of a stimulus, the GR is localized in the cytoplasm, where it binds as a monomer to the chaperone complexes containing HSP90 and HSP70. BCL-2-associated gene product-1 can act as a negative regulator, while HSP70-interacting protein acts as a positive one.^138^ Subsequently, HSP90 and HSP70/HSP90 organizing proteins attach to the complex, priming the opening of the steroid binding cleft of the GR, enabling GC binding.^139^ This GR/HSP90 heterocomplex is stabilized by protein 23 and several immunophilins like FK506-binding protein-51 (FKBP-51), FKBP-52 and cyclophilin D (CYP-40).^140^

Upon binding of GCs, GR conformational changes activate functional domains, including nuclear localization sequences within the hinge and LBD regions, binding of HSP70 and co-chaperone HSP40 that subsequently stimulates HSP70’s ATPase activity.^141^ HSP90 and CYP-40, together with FKBP-52, are involved in the transport of the GC–GR complex into the nucleus, while FKBP-51 has the opposite effect, keeping the complex in the cytoplasm,^138,142,143^ although it is not yet clear whether FKBP-51 has a specific effect in inhibiting the receptor or simply competes with GR-stimulating proteins such as FKBP-52.^144^

It was previously demonstrated that ALL cells expressed higher amounts of HSP90α compared with physiological blood cells.^145^ Nevertheless, it has been established that mRNA expression levels of HSP90, the HSP90/HSP70-based chaperone machinery, and co-chaperones do not correlate with GC resistance in pediatric ALL.^64^ Some hypotheses proposed to explain these findings are that the functional capacity of the co-chaperone molecules to form a complex with the GR is related to GC resistance (not the expression level of the co-chaperone molecule), or that the expression level of co-chaperones is important only upon GC exposure.^64^ In this regard, HSP90 inhibitors have been designed and used in ALL and other cancers and could be further exploited in the case of GC resistance.

We used reverse-phase protein arrays to demonstrate that LCK, a main regulator of TCR signaling, is hyperactivated in PPR patients. We showed that LCK inhibitors such as dasatinib, bosutinib, nintedanib, and WH-4-023 can induce cell death in GC-resistant T-ALL cells, and remarkably, cotreatment with dexamethasone is able to reverse GC resistance, even at therapeutic drug concentrations. This was confirmed by LCK gene silencing and ex vivo combined treatment of cells from PPR PDXs.^39^

An independent short-hairpin RNA-based screen using T-ALL cell lines and PDX samples also identified vulnerabilities in the pTCR/TCR pathway and a critical role for LCK in cell proliferation. Targeting LCK with dasatinib caused cell cycle arrest, and combination with dexamethasone resulted in significant drug synergy in preclinical models of T-ALL, including a murine study, designed like an early phase 2 human clinical trial, suggesting the clinical importance of this drug combination for high-risk T-ALL patients.^146^ ETP-ALL patients also present with hyperactivated LCK/calcineurin and mTOR/STAT3 axes and JAK/STAT and RAS/MAPK signaling pathways, and they could also benefit from cotreatment with LCK inhibitors.^78^ Moreover, the hyperactivation of mTOR enhances STAT3 and 40S ribosomal protein S6 phosphorylation, supporting the viability of cancer cells. This hyperactivation is most likely not caused by FLT3 mutations, but rather by an abnormal feedback loop through p70 S6K mutation. These findings have prompted researchers to target mTOR signaling using rapamycin or other inhibitors in GC-resistant leukemia.^46,147^ A recent work on a wide array of primary human T-ALL and PDX mice suggests that the combination of the mTOR inhibitor temsirolimus with dasatinib is even more efficacious in sensitizing GC-resistant T-ALL cells to dexamethasone.^112^

The role of deubiquitination in GC resistance was initially investigated a few years ago; the ubiquitin-specific protease USP9X is highly expressed in B-ALL contexts and its silencing impaired leukemic cell growth and induced apoptosis.^148^ Mechanistically, USP9X silencing leads to a decrease of MCL1, BCL2 and BCL-XL levels and an increase in BAX levels.^148^ Our teams further demonstrated that the complex of ubiquitin-specific proteases USP7 and USP11 deubiquitinates LCK and plays a critical role in LCK blocking GR activity.^149^ We observed that USP7 and USP11 are highly expressed in T-ALL and associated with poor disease prognosis. Leukemia progression is slowed down by USP11 ablation in vivo, but normal hematopoiesis is preserved. In this case, deubiquitination controls the activity and not the levels of LCK, preventing GR activation upon dexamethasone treatment. Indeed, impairment of USP7/11 or LCK activity led to increased *NR3C1* expression, chromatin and transcriptional changes in downstream genes, and sensitized cells to treatment with dexamethasone.^149^ This study underlined the role of deubiquitination in controlling resistance to therapy. For instance, it was shown that high expression of USP1 was correlated with poor prognosis in T-ALL patients. This upregulation of USP1 in the cell line CEM-C1 and in GC-resistant T-ALL patients seems to be guided by ALKBH5. Indeed, downregulation of ALKBH5 decreased the levels of USP1 and aurora B, increasing apoptosis via CEM-C1 cell sensitivity to dexamethasone, and reducing cell invasion.^150^

These studies highlight the significance of understanding the role of posttranslational modifications of the GR and its interaction with associated enzymes and show that focusing on GR-interacting proteins can have therapeutic implications for dexamethasone-resistant tumors.

### Transcriptional and chromatin properties of GR

The GR exhibits plasticity in the cytoplasm, as it responds to various signaling pathways and specificity with regards to the transcriptional output on chromatin.^6^ After translocation into the nucleus, GR associates with specific genomic GREs and sometimes forms a complex with other TFs and epigenetic modulators to activate or repress target genes (Figures 1 and 2).

There is interplay of GR with other TFs in a context-specific manner; direct protein-protein interactions with survival-promoting TFs result in GR sequestration, and thus restriction of the GC-initiated cell death-inducing activity of the GR.^151^ Vice versa, the mutual antagonism of GR and NF-κB or AP-1 has been proposed to contribute to the resistance to GC observed in different hematopoietic malignancies.^151^ However, the interaction of GR with other TFs such as STAT3 and STAT5 enhances the transcriptional activity of certain target genes.^152^ In immune cells, the liver receptor homolog-1 (LRH-1) and GR show opposing regulatory roles, with oncogenic properties and suppression of T-cell proliferation and T cell-mediated inflammation, respectively. Direct interaction between GR and LRH-1 regulates GC sensitivity in T-ALL, opening up possible perspectives to develop new therapeutic approaches for treating GC resistance.^153^ Estrogen-related receptor β (ESRRB) is another dexamethasone-inducible member of the ESRR family that binds to estrogen-related response elements. ESRRB silencing interferes with the expression of GR-controlled genes and results in GC resistance in vitro and in vivo, suggesting a critical GR partner that warrants further investigation in therapeutic treatment. The NOTCH1 target HES1 binds and suppresses the NR3C1 promoter.^63^ Elegant work by Real et al^154^ revealed that NOTCH1 inhibition can restore GC sensitivity in T-ALL via *NR3C1* reactivation. GCs can, in turn, inhibit γ-secretase inhibitor (GSI)-induced gut toxicity, providing a strong rationale for the clinical evaluation of combinations of GSIs and steroids for the treatment of human T-ALL. Li et al clearly demonstrated a link between neuroblastoma ras viral oncogene homolog and steroid resistance, while no difference was observed for vincristine or asparaginase sensitivity. Oshima et al revealed that KRAS-G12D mouse T-ALL tumors were specifically resistant to methotrexate instead of steroids and displayed increased sensitivity to vincristine treatment.^35^ Together, these findings suggest that better characterization of GR synergy with other TFs is necessary for the design of better treatments.

GR activity is controlled by epigenetic modulators; corepressor mutations in the nuclear receptor as well as increased expression of the enzymatically active members of the corepressor complex, histone deacetylases, have been described in relapsed ALL.^36,37^ NSD2 is a methyltransferase for lysine 36 on histone H3 frequently activated via mutations (p.E1099K) in relapsed T-ALL.^155,156^ GC-mediated transcriptional changes were inhibited in *NSD2*-mutant cells through repression of NR3C1 autoactivation via excessive H3K27me3 accumulation at the *NR3C1* promoter.^155^ Use of polycomb repressive complex 2 (PRC2) inhibitors restored *NR3C1* autoactivation by GCs and reversed GC resistance in vitro and in vivo. These findings suggest a new therapeutic approach in relapsed ALL cases with mutant *NSD2*. As mutations affecting members of the PRC2, such as EZH2, SUZ12, and EED, have been identified in T-ALL,^157^ better integration of PRC2 and NSD2 mutations and activity must be performed in PPR ALL cases. Finally, we and others have shown that the JMJD3 demethylase is induced by NFκB and plays the role of NFκB and NOTCH1 partner in T-ALL via removing the H3 lysine 27 methyl marks, leading to gene derepression.^158^ In light of the competition between NOTCH1 and NFκB pathways with GR, use of JMJD3 inhibitors, such as GSKJ4, could be used to suppress oncogenic signaling pathways and induce sensitivity to GCs.

### The role of chromatin modulators and chromatin structure in GR transcriptional programs

With the advent of chromatin analysis technologies, such as the assay for transposase-accessible chromatin sequencing or chromatin conformation capture technologies (such as HiC), new levels of gene regulation by GR have been uncovered. Most of the GR binding (up to 95%) happens at the level of constitutively accessible chromatin, open before hormone treatment, with a few GR binding events taking place at sites with hormone-induced remodeling (eg, nucleosomal sliding or eviction).^159^ At most sites, TFs induce remodeling of the chromatin landscape to maintain high levels of accessibility and facilitate the recruitment of TFs, such as GR.^160^ Some studies, for instance, showed that AP-1 maintains some chromatin sites in an open conformation and facilitates the selectiveness of GR by colocalizing to the same regulatory elements in a high (51%) fraction of GR binding sites.^161^GR interacts with histone acetyltransferases, such as cAMP response element-binding protein (CBP)^162^ and P300,^163^ and it can also bind to the insulator-binding CCCTC factor (CTCF) to alter chromatin structure.^164^ Mutational analysis of 71 diagnosis–relapse cases and 270 ALL cases that did not relapse identified CBP mutations in 18.3% relapsed cases.^37^ The mutations are projected as loss of function, resulting in truncated proteins or abolishing the histone acetyltransferase activity of CBP and activation of GR targets. Use of histone deacetylase inhibitors leads to partial activation of GR target and might synergize with GC to push ALL cells toward apoptosis.^37^

Several GR-related transcriptional activities were studied using the MMTV promoter system. Activation of the MMTV promoter requires GR-mediated chromatin-remodeling through recruitment of BRG1, a component of the switch/sucrose non-fermentable (SWI/SNF) chromatin-remodeling complex.^165^ Using high-intensity UV cross-linking in vitro that detects rapid interactions between protein factors and DNA, it was shown that GR and BRG1 binding are transient and occur in an oscillatory manner depending on SWI/SNF-mediated ATP hydrolysis.^166^ Recent studies demonstrated that in certain genomic loci, GR binding site accessibility is independent of the presence of the SWI/SNF complex, suggesting that additional remodelers must be involved in these cases.^159^

Long-distance chromosomal interactions are critical for bringing into proximity the promoter of GR and its response genes.^167,168^ GREs are located at a 50 bp to 100 kb distance from the transcriptional start sites of the genes they control.^168^ Chromatin conformation capture-based technologies, such as HiC coupled to chromatin immunoprecipitation focusing on enhancer marks (histone 27 acetylation), showed an increase in connectivity between enhancer elements bound by GR and ESSRB in human T-ALL cells^8^ and target gene promoters upon dexamethasone treatment. BIM, a proapoptotic member of the BCL2 family, is upregulated upon GC stimulation and antagonizes antiapoptotic members such as BCL2, BCL-XL, and MCL-1.^169,170^ Moreover, a critical role for BCL2 family proteins have already been identified in GC-induced apoptosis of malignant lymphocytes.^38^

**Figure 5.**
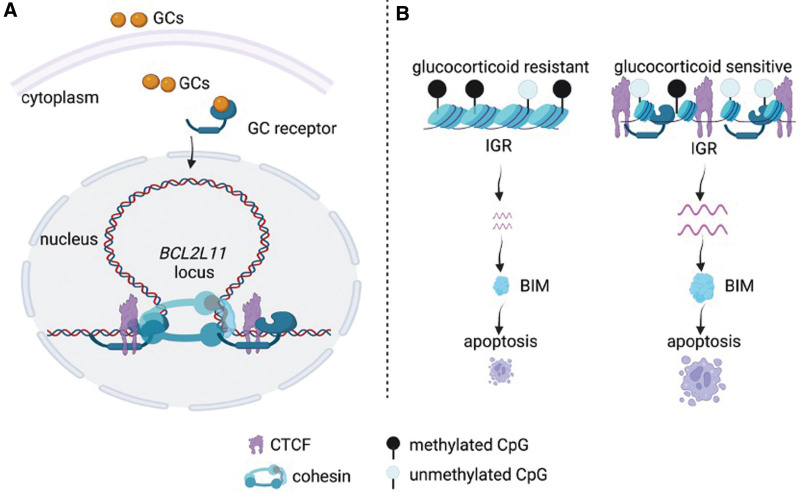
**Lessons from *BCL2L11 (BIM*), a well-characterized transcriptional target of GR**. (A) Upon binding to GCs, GR translocates into the nucleus and to the BIM promoter (left side of the stem loop) and IGR enhancer (right side of the stem loop). (B) IGR chromatin structure and epigenetic marks are shown in GC-resistant vs sensitive ALL samples. Lower DNA methylation in GC-sensitive samples facilitates GR and CTCF binding upon GC application. ALL = acute lymphoblastic leukemia; CTCF = CCCTC factor; GC = glucocorticoids; GR = glucocorticoid receptor.

Focusing on DNA hypersensitive sites, Jing and colleagues^171^ identified 18,777 sites that differ in lymphocytes compared with other tissues, including 11,101 genomic regions with lymphocyte-specific open chromatin structure (LSO) and 7676 with lymphocyte-specific closed chromatin structure. The proapoptotic BIM gene had an LSO located 36.5 kb downstream of its promoter, an area called **IGR** that acts as a BIM enhancer (Figure 5).^170^ Further analysis demonstrated that increased CTCF and GR binding at the BIM intronic region (IGR) was lymphocyte specific. GR-sensitive cases present with an accessible (open) IGR locus and lower DNA methylation at this locus, compared with GR-resistant tumors.^171^ This conformation permits the binding of the GR and the insulator protein CTCF to enhance BIM transcription, contributing to the acute sensitivity of normal and malignant lymphocytes to GC-induced apoptosis.^171^ Thus, this lymphocyte-specific chromatin conformation can determine GC resistance in ALL. The GR can cooperate with CTCF at the level of genome-wide LSOs and mediate the formation of a transcriptionally active DNA loop to trigger gene transcription. On the contrary, this loop is inhibited by increased DNA methylation in GC-resistant ALL and nonlymphoid cell types.^171^ Research conducted by our groups showed that treatment with USP7 inhibitors enhances the binding of GR to BIM IGR and increases chromatin accessibility and enhancer-promoter looping.^149^

## CONCLUSIONS

Ongoing research suggests that a better understanding of GR nuclear functions and cofactors in ALL and use of inhibitors against epigenetic cofactors or enzymes controlling posttranslational modifications in combination with GCs could be of therapeutic value in high-risk ALL cases. The rationale for this is the following: (1) a number of ALL oncogenes are TFs, and ALL present with frequent mutations or aberrant activity of epigenetic modulators; (2) GR presents with plasticity with regards to signal integration from multiple pathways and specificity with regards to transcriptional target activation or repression, suggesting that focusing on the nucleus and epigenetics will be more beneficial from a therapeutic stand-point; and (3) all the pathways targeted by inhibitors of TFs such as LCK or NOTCH1 reflect transcriptional programs that act in cooperation with, competition with, or in parallel to GR signaling. Additionally, (4) GR protein is highly posttranslationally modified, is controlled by chaperones, and has many interacting proteins. At the same time, as discussed earlier, there are no common gene expression changes observed amongst different dexamethasone-resistant relapsed PDX models, suggesting that mechanisms of resistance might differ among individuals.^60^ This brings into perspective the protein levels and modifications of GR and associated members of the pathway and other potentially antagonizing pathways.

Thus a personalized therapy approach should take into consideration the following parameters at diagnosis and post the first week of treatment with GCs: (1) the mutational status of NR3C1, including the exact type and the homozygous or heterozygous presence of mutations, as well as NR3C1 cofactors that have been shown to be mutated in ALL, such as CBP; (2) the mutational status of pathways such as NOTCH1 and PTEN/AKT/PI3K; (3) the transcriptional levels and isoforms of NR3C1; (4) levels of LCK, USP7, and USP11; (5) expression of proapoptotic factors. This will be coupled to (6) detection of minimal residual disease and clustering of the patient to standard- or high-risk groups. Technologies such as Reverse-Phase Protein Array (RPPA) and next-generation sequencing allow us to quickly and efficiently assess these clinical parameters and prioritize therapies and downstream actions accordingly. Refractory/relapsed patients, where mechanisms of resistance exist, will be especially benefited by this approach.

For instance, recent work uncovered unexpected roles for the LCK kinase and its regulation of downstream TCR signaling in suppressing apoptosis and driving continued leukemia growth in competition with GCs, as described earlier.^39,149^ This justifies efforts to sensitize leukemia cells to GC response with LCK inhibitors such as dasatinib, bosutinib, and nintedanib both in T-ALL and BCP-ALL,^39,79,146^ as well as with dasatinib derivatives coupled to a proteolysis-targeting chimera approach against LCK.^172^The LCK inhibitor dasatinib in combination with mTORC1 inhibition induced potent T-ALL cell death through reducing MCL-1 protein expression.^112^ Similarly, the use of deubiquitinase inhibitors together with GCs might also hold potential for targeting aggressive ALL cases. Future research focusing on better understanding GR participation in complexes and its modes of regulation is warranted.

## ACKNOWLEDGMENTS

We thank members of our groups for providing feedback on the article. Figures were created using Biorender (https://www.biorender.com/).

## AUTHOR CONTRIBUTIONS

CB, TP, VS, and PN wrote the article.

## DISCLOSURES

The authors have no conflicts of interest to disclose.

## SOURCES OF FUNDING

The Ntziachristos laboratory is supported by the Research Foundation Flanders (FWO, G0F4721N), start-up funds from the Department of Biomolecular Medicine, Ghent University, a Flanders interuniversity consortium grant (BOF.IBO.2023.0006.02) and a Cancer Research Institute Ghent (CRIG) partnership grant. VS was supported by the co-financing of the European Union - FSE-REACT-EU, PON Research and Innovation 2014–2020 DM1062/2021 and by Fondazione Associazione Italiana per la Ricerca sul Cancro (AIRC, MFAG 2018, ID. 21771).
